# Discovery of BVDU as a promising Drug for autoimmune diseases Therapy by Dendritic-cell-based functional screening

**DOI:** 10.1038/srep43820

**Published:** 2017-03-08

**Authors:** Shuai Chen, Jinfeng Zhou, Yingying Cai, Xinyuan Zheng, Sirong Xie, Yuhan Liao, Yu Zhu, Chaoyan Qin, Weiming Lai, Cuixia Yang, Xin Xie, Changsheng Du

**Affiliations:** 1 Department of Central Laboratory, Shanghai Tenth People’s Hospital of Tongji University, School of Life Sciences and Technology, Tongji University, Shanghai 200092, China; 2 State Key Laboratory of Drug Research, Shanghai Institute of Materia Medica, Chinese Academy of Sciences, Shanghai 201203, China; 3 Shanghai Key Laboratory of Signaling and Disease Research, School of Life Sciences and Technology, Tongji University, Shanghai 200092, China

## Abstract

Dendritic cells (DCs) play a critical role in the pathogenesis of autoimmune diseases including multiple sclerosis, and targeting DCs’ cytokines production is an important strategy for autoimmune diseases treatment. By establishing a high-throughput screening system, we analyzed LOPAC drug library to identify drugs that control the secretion of IL-6 by DCs, we selected the most likely candidate drug, BVDU, and found that it affected not only IL-6 production, but also that of IL-12, IL-1β during the DCs differentiation and maturation. The mechanism studies showed that BVDU treatment restricted the phosphorylation of MAP kinase, which played an important role in DC cytokine production. We further assessed the *in vivo* therapeutic potentials of BVDU on mouse models including EAE and STZ-induced T1D, and found that BVDU treated EAE mice exhibited significantly lower EAE clinical scores, decreased leukocyte infiltration in central nervous system lesions, and reduced demyelination. As in T1D mice, BVDU treatment also showed promising therapeutic effects based on both alleviated disease symptoms and tissue pathogenesis. More interestingly, the modulating effect of BVDU on IL-6 production was further verified in human primary DCs. The above data supported the promising application of our screen model, and also the potential of BVDU for autoimmune diseases therapy.

MS, multiple sclerosis, is a demyelinating and neurodegenerative disease of the central nervous system (CNS) in which immune dysregulation leads to loss or deficit of sensory, motor, autonomic, and neurocognitive function. This disease occurs with a prevalence between 2 and 150 per 100000 in adults between 20 to 40 years of age^1^. MS leads to an enormous socioeconomic loss second only to trauma in young adults^2^,^3^.

CD4^+^ T cells are considered to have a role in the initiation of MS by mediating autoimmunity in the early stage of the disease^4^,^5^. Th1 cells, have long been accepted as one of the main effector cells which mediate the pathogenesis of MS. Researchers have subsequently found that there are IL-17-expressing T cells in lesions of brain tissues isolated from patients with MS^6^, and mice which lack Th17 cells are resistant to suffer from the disease in animal models^7^,^8^, both of which indicate that Th17 are also involved in the disease and play a critical role in the progress.

Dendritic cells capture antigens in the periphery, migrate to lymph node, present antigens and secrete cytokines to initiate T cells differentiation^9^. Substantial research shows that some among these cytokines, TGF-β and IL-6, are required in the differentiation of Th17 cells^10^,^11^,^12^. IL-6 orchestrates a series of cytokine dependent signaling pathways to amplify Th17 cell differentiation^13^.

Researchers have reported that they developed a dendritic cell screening system by engineering DCs to steadily express a fluorescent marker gene under the control of the IL-1β promoter^14^,^15^,^16^. We have developed and validated a high-throughput screening assay utilizing a specific fluorogenic substrate and IL-6 secretion by DCs to search for new drugs targeting at IL-6 secretion in DCs. This assay was applied to screen the LOPAC library containing 1,280 synthetic compounds. The drug with most promising potential, BVDU, was picked out for further therapeutic analysis on autoimmune mouse models including EAE and T1D. We found that BVDU showed promising therapeutic effects based on both alleviated disease symptoms and diminished tissue pathogenesis. Furthermore, the modulating effects of BVDU on IL-6 production were verified in human primary DCs.

## Results

### Development and optimization of DCs based screening assay for IL-6 secretion modulators

**Figure 1:**
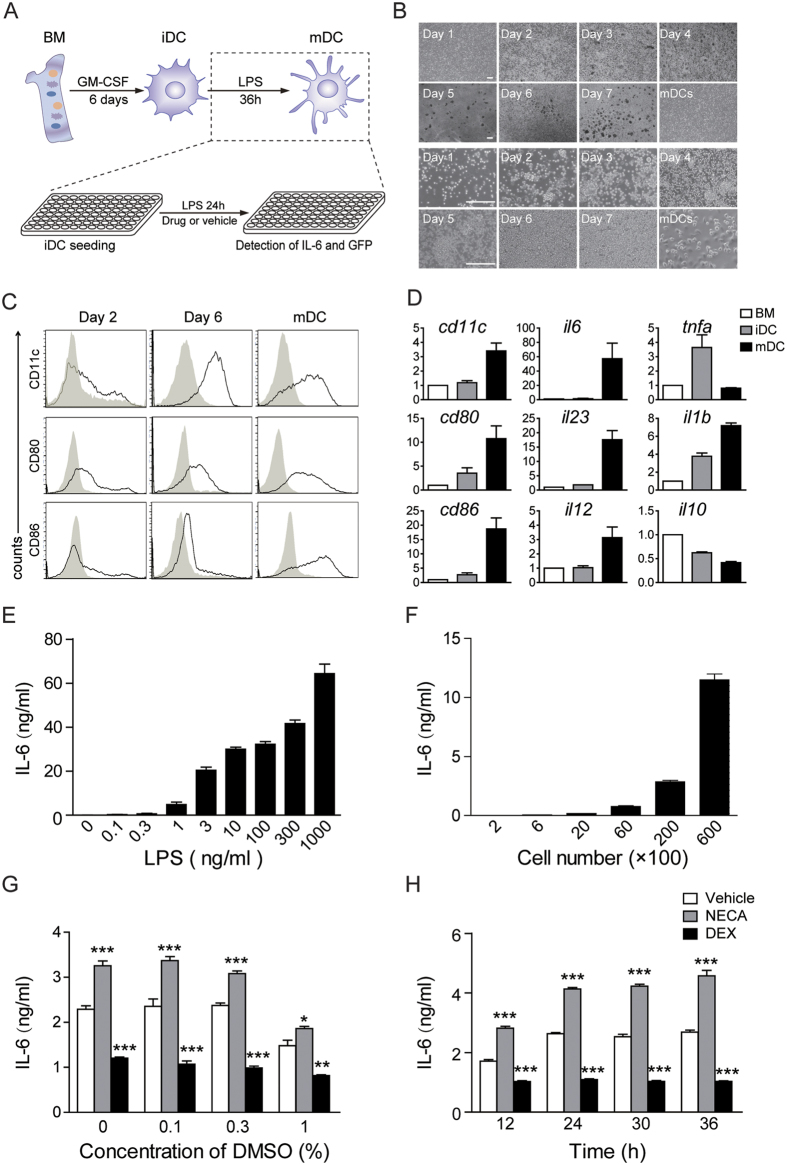
Development and optimization of a DC based assay for screening IL-6 modulators. (**A**) The experiment protocol for DCs development *in vitro* and IL-6 modulators screening. (**B**) Morphology of bone marrow cells (BM), immature DCs (iDC) start from day 2 after GM-CSF treatment) and mature DCs (mDC). Scale bars: 200 μm. (**C**) iDC (day 2 and day6) and mDC were examined for the surface expression of CD11c, CD80 and CD86. (**D**) Relative mRNA levels of *cd11c, cd80, cd86, il6, il12, il23, il1b, tnfa*, and *il10* in BM cells, iDC, and mDC. (**E**) Optimization of LPS concentration for mDCs induction. (**F**) Optimization of cell numbers seeded in 96-well plate for drug screening. (**G**) Optimization of drug vehicle (DMSO) concentration. NECA and dexamethasone (DEX) were used as positive control. (**H**) Optimization of incubation time. Data are mean ± SEM (n = 3). ***p < 0.001 versus vehicle.

DCs constitute a very low proportion of leukocytes *in vivo* and functional analysis of DCs is limited by low cell yields. In order to obtain a large amount of DCs to perform high-throughput screening for IL-6 secretion modulators, *in vitro* DCs generation system was set up and optimized. GFP expressing bone marrow cells were collected from a GFP transgenic mouse, immature DCs (iDCs) were induced by adding GM-CSF for 6 days, and with a further stimulation of LPS for 36 hours, we got matured DCs (mDCs) (Fig. 1A). During this process, we checked for morphology changes. Bone marrow cells were separated. The small cell clones were visible from day 2, and large clones emerged on day 6 with the development of the cells, and the clone morphology remained unchanged from iDCs to mDCs (Fig. 1B). In order to characterize the *in vitro* generated DCs, the expression of DCs surface markers were examined, and the results showed that CD11c expression was slightly induced at an early stage on day 2, but almost no CD80 or CD86 expression was detected as they are markers of mature DC (mDCs)^17^, while on day 6, CD11c was highly induced, CD80 and CD86 were moderately induced; in mDCs, all CD11c, CD80, and CD86 expression were highly induced (Fig. 1C). These data was in accordance with previous reports and indicated that the obtained DCs are adequately suitable for subsequent experiments. mRNA expression of DCs markers was further analyzed. As shown in Fig. 1D, *cd11c, cd80, cd86*, and *il1β* were gradually upregulated from BM to mDCs, and *il6, il12, il23* maintained a low expression level from BM to iDCs, but were significantly upregulated from iDCs to mDCs. However *il10* was gradually decreased from BM to mDCs. Interestingly, *tnfα* was firstly upregulated from BM to iDCs, and then decreased from iDCs to mDCs, these data further support the normal function of the *in vitro* generated DCs.

In order to develop a suitable system for IL-6 modulators screening, various experiment conditions, including LPS stimulation concentration, cell number, vehicle (DMSO) concentration and incubation time were optimized. As shown in Fig. 1E, a clear dose response existed between the IL-6 production and LPS concentration, in addition, with the increase in cell numbers, IL-6 secretion increased dramatically (Fig. 1F). The vehicle (DMSO) concentration and the incubation time were found to significantly affect IL-6 secretion, however lower concentration of DMSO and longer incubation time showed a larger signal window (Fig. 1G and H). Based on the above observations, we eventually chose the following conditions: LPS stimulation was 10 ng/ml, cell number was 10,000 cells per well, DMSO concentration was 0.1% and incubation time was 24 hours for further screening of IL-6 modulators in 96-well plates.

### Assay performance

**Figure 2:**
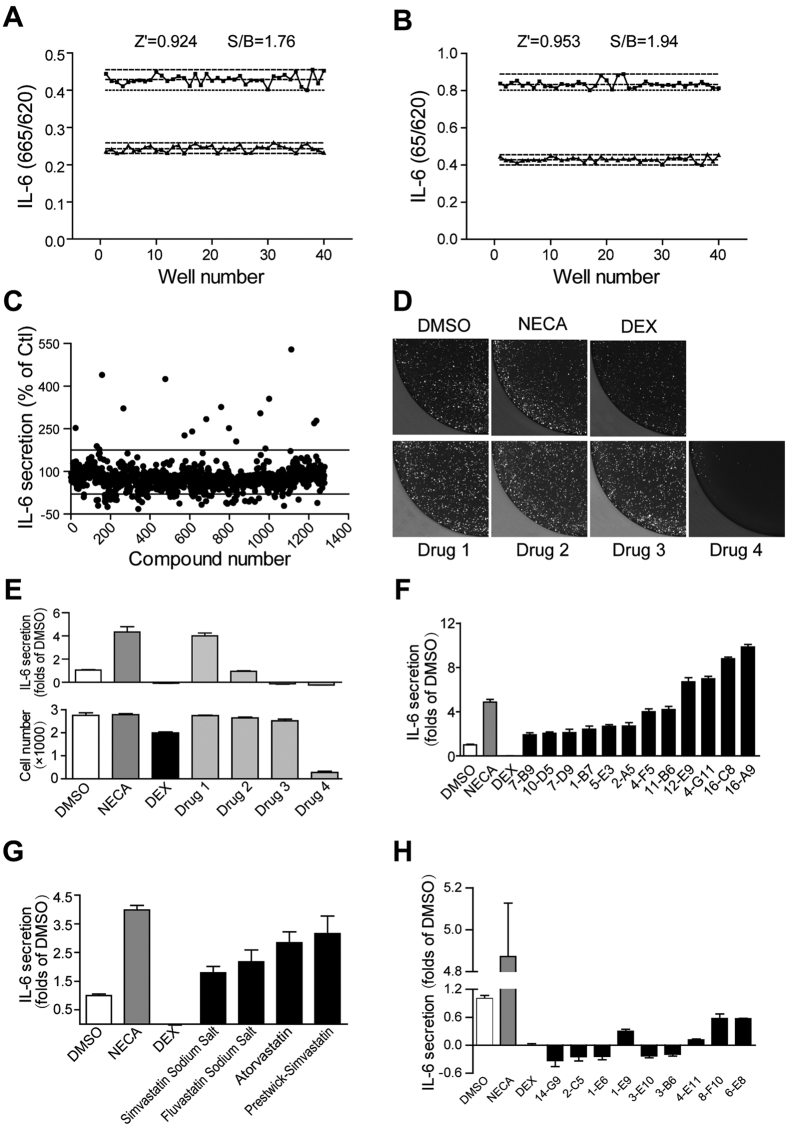
Screening IL-6 modulators in the LOPAC compounds library with the DC based assay. The assay performance was analyzed with positive controls by Z’ factor, of 10 μM NECA (**A**), of 100 nM DEX (**B**), dashed lines indicate means ± SD of 48 data points. Result of the primary screening of 1280 compounds in duplicate setup with DC screening assay (**C**). Cell number changes were detected by GFP signal after drug treatment (**D**). (**E**) Quantitation of the cell numbers (bottom panel) and IL-6 secretion (top panel) in the relative wells present in (**D**). (**F**) Compounds which significantly increased IL-6 secretion in the primary screening were further verified in triplicates. (**G**) Verification of simvastatin sodium salts on IL-6 secretion. (**H**) Compounds which inhibited IL-6 secretion were further verified in triplicates. NECA (10 μM) and DEX (100 nM) were used as the relative positive controls.

The Z’ factor is the normalized standard deviation window between the negative controls and positive controls. It is widely used for the evaluation of High-throughput screening (HTS) assay quality. In general, a Z’ value above 0.5 suggests that an assay is suitable for HTS^18^. The Z’ values of our assay were 0.92 and 0.95 for the enhancers and inhibitors respectively (Fig. 2A and B). The signal/background (S/B) ratio is another metric used to evaluate the assay window and the S/B ratio in our assay was 1.76 and 1.94 (Fig. 2A and B). These data indicated that the system was adequately optimized with high quality for HST. Then the assay was applied in the screening of a compound library of LOPAC (Library of Pharmacologically Active Compound, Sigma) consisting of 1280 compounds^18^. In the primary screening, 1280 compounds were tested at 10 μM in a duplicate setup, with NECA and DEX as positive controls, and DMSO as a negative control. A small portion of the candidates did show obvious effects on IL-6 production (Fig. 2C). In order to exclude false effects caused by changes in the number of cells, we monitored cell numbers at the end of the screening in each well by measuring the GFP signal with high-throughput fluorescent microscopy (Fig. 2D and E), since cells were derived from GFP transgenic mice.

Positive drugs DEX (100 nM) and NECA (10 μM) could significantly modulate IL-6 production without influencing cell numbers (Fig. 2D and E). The compounds in the library could also be divided into four groups according to their effects on IL-6 production and cell survival: the first group enhanced IL-6 production but didn’t change the cell numbers (such as Drug 1), the second group didn’t change the cell numbers and IL-6 production (most of the candidates, such as Drug 2), the third group consisted of those candidates which changed IL-6 production but didn’t affect cell numbers (a small portion of the candidates which were the targets of our screening, such as Drug 3), while artificial candidates in the fourth group did change cell numbers (such as Drug 4) (Fig. 2D and E).

Finally, from the screening, we found 12 compounds which enhanced the secretion of IL-6 by DCs (Fig. 2F, Table 1), among which, Podophyllotoxin, Colchicine, Vinblastine sulfate salt and Vincristine sulfate are the inhibitors of microtubule assembly, significantly increased DCs’ IL-6 secretion. Also 2-Chloroadenosine and Metrifudil, which are Adenosine receptor agonists, were found to enhance DCs IL-6 secretion. Mevastatin, which inhibits post-translational prenylation of proteins such as Ras and geranylgeranylation of Rho, was found to increase DCs IL-6 secretion in this library (Fig. 2F). Later we tested some Statins drugs from other compound libraries and we found that this family had the same effects on increasing the secretion of IL-6 by DCs (Fig. 2G). On the other hand, nine compounds which had significant effects on reducing DCs’ IL-6 secretion were picked out, and most of these compounds had not yet been reported to decrease IL-6 secretion (Fig. 2H, Table 2).

### BVDU reduces IL-6 production

**Figure 3:**
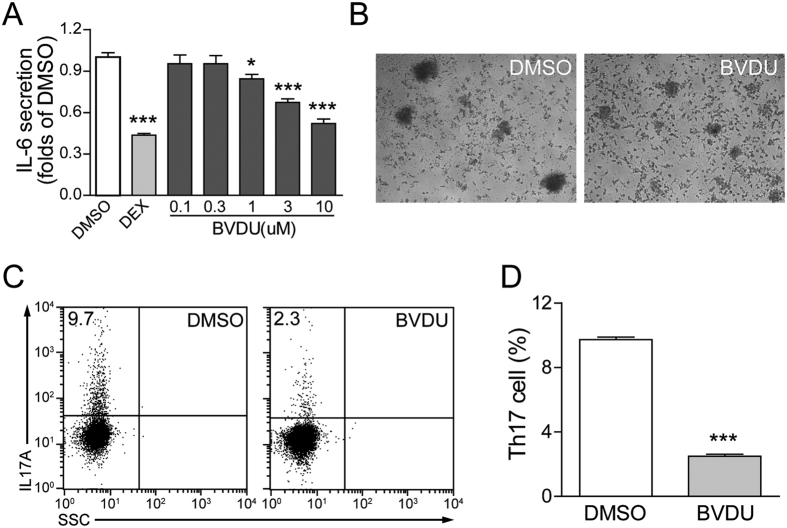
BVDU treatment reduces IL-6 production and Th17 differentiation in DC–T cell co-culture system. (**A**) Dose effects of BVDU on IL-6 production in mDCs. (**B**–**D**) BVDU regulates Th17 differentiation in the *in vitro* DC–T cell co-culture system, the clone size was significantly reduced upon BVDU treatment (**B**), representative pictures of FACS analysis (**C**) and statistics data (**D**) were shown. Data are mean ± SEM (n = 3). *p < 0.05, ***p < 0.001 versus DMSO.

After two turns of screening, one drug named BVDU showed a promising inhibitory effect on IL-6 production by DCs; we further analyzed its characteristics on IL-6 production and DCs development. Firstly, dose effects of BVDU on DCs’ IL-6 production were analyzed, and the results showed that 1 μM BVDU can obviously inhibit IL-6 production, and more significant effects were observed at higher doses of 3 μM and 10 μM, while lower doses of 0.1 μM and 0.3 μM showed no effects (Fig. 3A).

As previously reported, many autoimmune diseases such as multiple sclerosis are initiated by the peripheral immune system. During pathogenesis, driven by cytokines secreted by antigen presenting cells (mainly by DCs), CD4^+^ T cells are abnormally activated and differentiated into T helper cells (Th1 and Th17), migrate to the injured tissues and promote disease pathogenesis^19^. IL-6 is a pro-inflammatory cytokine critical for Th17 development^13^ and *in vitro* Th17 differentiation can be achieved by stimulating naive T cells with anti-CD3/CD28 Abs in the presence of IL-6 and TGF-β. We mimicked *in vivo* T cell differentiation by co-culturing of DCs with naive T cells as previous mentioned^20^. Th17 cell percentage was monitored by intracellular staining of IL-17, specific Th17 marker, and at the end of the *in vitro* differentiation, smaller T cell clones were observed in the BVDU treated group, compared to the vehicle (Fig. 3B). FACS analysis of the co-cultured cells demonstrated that BVDU treatment significantly decreased Th17 percentage (Fig. 3C and D). These data indicated that BVDU can inhibit IL-6 production by DCs, which further led to the inhibition of Th17 cell differentiation.

### Impact of BVDU on DCs’ differentiation, maturation and cytokine production

**Figure 4:**
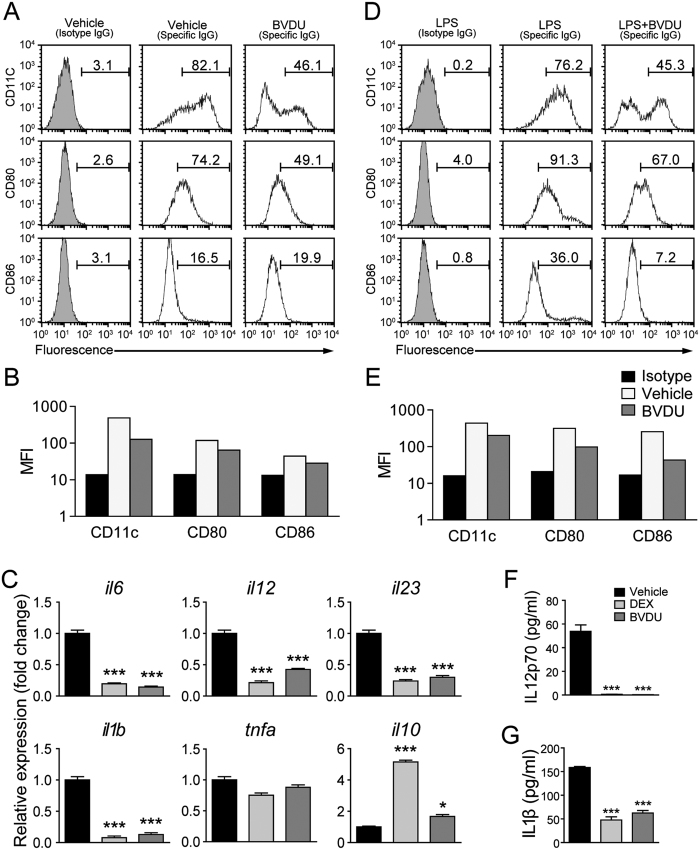
Effects of BVDU on DC differentiation, maturation and cytokine prouction. (**A**–**C**) Bone marrow cells were cultured with GM-CSF and IL-4 for seven days with or without BVDU, then harvested for CD11c, CD80, and CD86 staining and flow cytometry analysis (A and B) or qPCR analysis of relative expression levels of *il6, il12, il23, il1b, tnfa, il10, cd11c, cd80*, and *cd86* with the dexamethasone treatment as positive control (**C**). (**D**–**G**) Bone marrow cells were cultured with GM-CSF and IL-4 for seven days, BVDU was added from day 5, and LPS were added from day 7 and maintained for 36 hours, then cells were harvested for CD11c, CD80, and CD86 staining and FACS analysis (**D** and **E**), at the same time, the supernatants of the cultures were harvested for ELISA analysis for cytokine production (**F** and **G**). Data are mean ± SEM (n = 3). *p < 0.05, ***p < 0.001 versus DMSO.

To determine if BVDU stimulation influenced the differentiation of DCs, bone marrow cells were treated with 10 μM BVDU and analyzed by FACS. The results showed that BVDU treatment significantly decreased both CD11c and CD80 positive percentages (Fig. 4A). At the same time, the mean fluorescence intensity of CD11c, CD80, and CD86 were also slightly decreased upon BVDU treatment (Fig. 4B). The mRNA expression level of the DCs related cytokines were further quantified, and we found that *il6, il12, il23, il1β*, were significantly down-regulated when treated with BVDU. However, *tnfα* expression was not significantly influenced by BVDU. The anti-inflammatory cytokine gene *il10* expression was upregulated 2 folds when treated with BVDU. BVDU exhibited an effect almost identical to that of the positive control, Dexamethasone (DEX) (Fig. 4C).

We also investigated BVDU’s influence on LPS-induced maturation of DCs. DCs were treated with LPS alone, or LPS + BVDU. Adding of LPS to the culture induced significantly higher expression of CD80 and CD86 as compared to immature DCs. Also, BVDU treatment significantly inhibited LPS-induced expression of CD80, CD86 and CD11c at both the percentage and the MFI level (Fig. 4D and E). The protein level of IL-12p70 and IL-1β in the supernatant was further analyzed by ELISA, and the results verified that BVDU treatment significantly inhibited both IL-12p70 and IL-1β production by DCs (Fig. 4D). This data demonstrated that BVDU-treated DCs still expressed low levels of CD80, CD86 and CD11c after LPS stimulation. BVDU maintained DCs in an immature state.

### BVDU suppresses MAPK and NF-*κ*B pathways in LPS stimulated DCs

**Figure 5:**
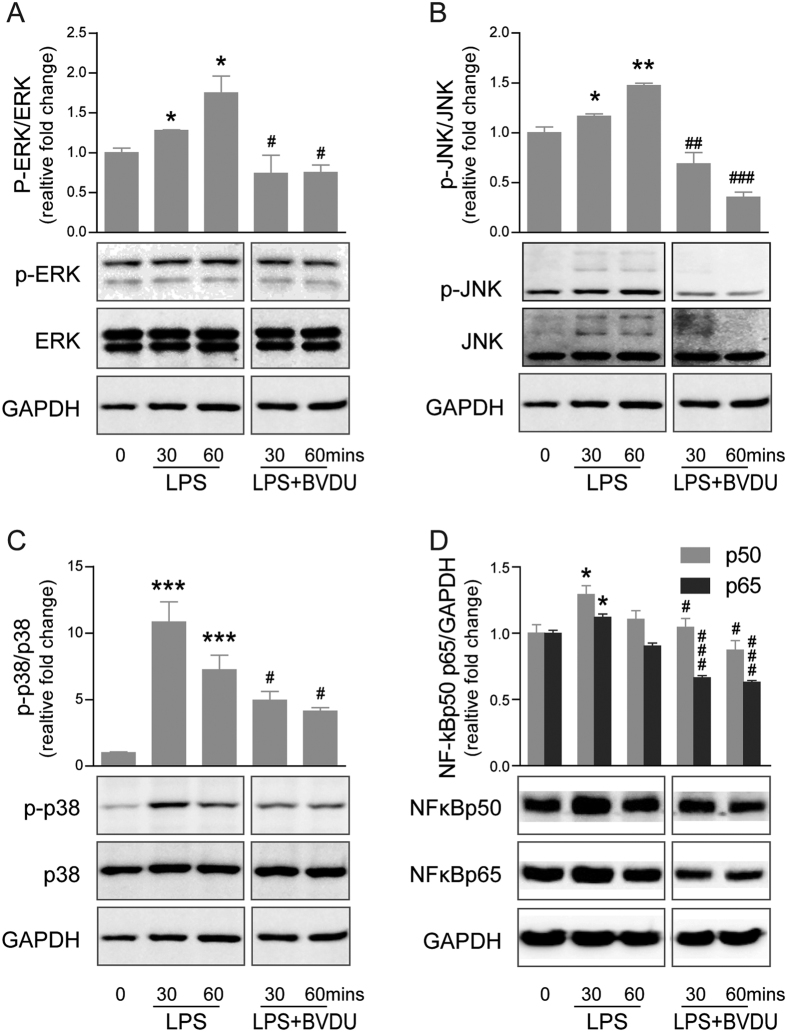
BVDU treatment inhibits of MAPK and NF-κB activity in DCs. iDCs were stimulated with 100 ng/mL LPS with or without BVDU (10 μΜ) and cells were harvested and lysed at the indicated time points for further immunoblotting analysis. (**A**–**C**) The protein levels of total or phosphorylated forms of ERK, JNK and p38 MAPK were analyzed by relative antibodies. (**D**) The protein levels of NF-κB p65 and p50 were determined by Western blot with relative antibodies. GAPDH are shown as a housekeeping protein control. The statistics data were shown at the top panel of each blot figure normalized by GAPDH. The data are representative of three independent experiments showing similar results. Data are mean ± SEM (n = 3), *p < 0.05, **p < 0.01, ***p < 0.001 versus iDC without LPS or BVDU, ^#^*p* < 0.05, ^##^*p* < 0.01, ^###^*p* < 0.001 versus iDC with LPS treatment at the indicated time point.

The activation of MAPKs and NF-*κ*B is crucial for DCs maturation and the inflammatory response^21^. LPS stimulation of TLR-4 signaling activates MAPKs and NF-*κ*B signal pathways, resulting in DCs maturation^22^. To explore the molecular mechanisms of the BVDU inhibitory effect, we determined whether MAPKs and NF-*κ*B activation were altered by BVDU treatment in LPS-stimulated DCs. Similar with what was previously reported, we observed that LPS stimulation for 30 mins or 60 mins significantly enhanced the phosphorylation of all three MAPKs including ERK, JNK, and p38 in DCs. More interestingly, when the DCs were further treated with BVDU, the phosphorylation of these three MAPKs was significantly decreased (Fig. 5A–C).

As NF-*κ*B signaling is another downstream pathway activated by LPS, we also analyzed the effect of BVDU on this pathway by quantifying the expression of p65 and p50, the important component of NF-*κ*B signaling pathway and the results indicated that LPS treatment increased the expression level of p65 and p50, however, BVDU treatment significantly inhibited p65 and p50 expression (Fig. 5D), these data indicated that BVDU treatment could suppress MAPK and NF-*κ*B pathways in LPS stimulated BMDCs, which further affected DCs maturation and cytokine production.

### BVDU ameliorates clinical symptoms and CNS infiltration in EAE

**Figure 6:**
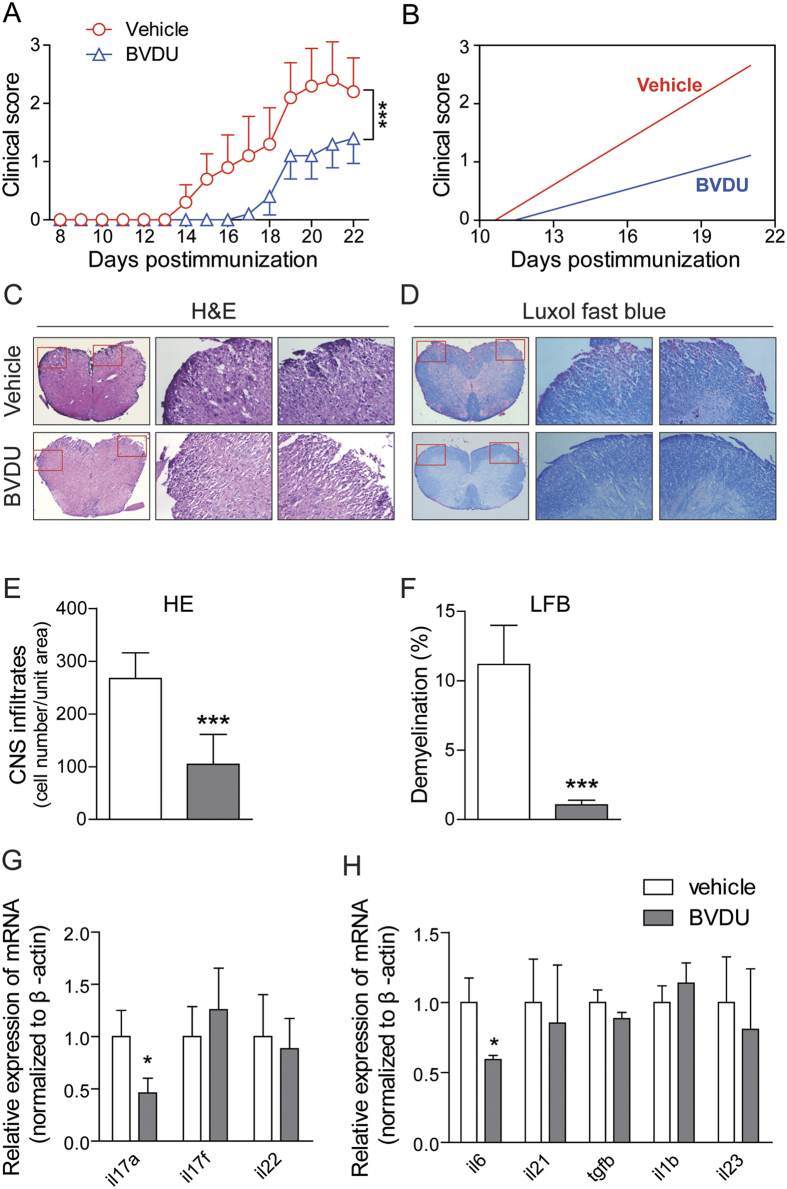
BVDU alleviates clinical symptoms of EAE. (**A**) Clinical scores of mice treated with BVDU (20 mg/kg) or vehicle (saline) once daily via oral administration from days 3 to the end of the EAE induction. Data are means ± SEM. ***p < 0.001 versus vehicle (two-way ANOVA test). (**B**) Linear regression analysis of the clinical scores of mice treated with BVDU or vehicle. (**C**,**D**) H&E and Luxol fast blue staining of paraffin sections of spinal cords isolated from vehicle or BVDU (20 mg/kg) treated EAE mice on day 24. (**E**,**F**) Quantification of CNS infiltrates and the amount of demyelination presented in (**C**) and (**D**). (**G**,**H**) qPCR analysis of relative expression levels of *il6, il21, tgfb, il1b, il23, il17a, il17f, il22* in splenocytes isolated from EAE mice treated with BVDU (20 mg/kg) or vehicle on day 10 after EAE induction. Data are mean ± SEM. Three mice from each group were sacrificed, and 20 sections from each mouse were analyzed. *p < 0.05, ***p < 0.001 versus vehicle.

Since our data demonstrated that BVDU could inhibit IL-6, IL-12, IL-23 and IL-1β production in DCs, and these pro-inflammation cytokines played a critical role in initiating the autoimmune disease, we hypothesized that this drug might have promising potential in treating autoimmune disease. To further verify this hypothesis, an EAE mouse model of multiple sclerosis was constructed as previously described^23^, BVDU was given orally once daily from day 3 post immunization until day 24, whereas the control mice were administrated with 0.9% saline. The data showed that in control group, the clinical symptoms initiated on day 13, and gradually increased until the end of the experiment, and in BVDU treatment group, disease initiated at later stage of the day 16, and more interestingly, the disease severity was also significantly alleviated (Fig. 6A and B). As multiple sclerosis is initiated in peripheral immune system and the disease lesions happened in central nerves system, the immune cell (leukocytes) infiltration into the spinal cord is another important index of disease severity, histological examination of spinal cord was performed on day 24 post immunization. In the vehicle treated mice, there existed a huge number of infiltrating leukocytes, whereas BVDU treatment caused a dramatic decrease of leukocyte infiltration in spinal cord (Fig. 6C and E). Demyelination of axon fibers is another important pathological change in EAE. So Luxol fast blue staining was performed to check the demyelination status in EAE mice. The results showed multiple widespread areas of myelin damage in white matter region of the spinal cord in saline-treated EAE mice. Whereas in BVDU-treated animals, such demyelination phenomenon was greatly reduced (Fig. 6D and F). Taken together, these data indicated that BVDU reduced EAE severity accompanied by decreased CNS inflammation and demyelination, which indicated a promising potential of BVDU in multiple sclerosis or possibly other autoimmune diseases treatment in the future. As our above data demonstrated that BVDU treatment inhibited IL-6 production, we further examined the IL-6, TGF-β, IL-1β, IL-21 and IL-23 expression level in EAE mice splenocytes and found that BVDU treatment significantly inhibited the IL-6 expression level but did not change that of TGF-β, IL-1β, IL-21 and IL-23 (Fig. 6H). As IL-6 facilitates the differentiation of Th17 cells, one of the major pathogenic players in MS, we also detected the expression level of IL-17A, IL-17F, and IL-22, the marker genes of Th17, and the results showed that IL-17A was significantly decreased upon BVDU treatment, but IL-17F and IL-22 were not affected (Fig. 6G).

### BVDU ameliorates clinical symptoms of other autoimmune diseases

**Figure 7:**
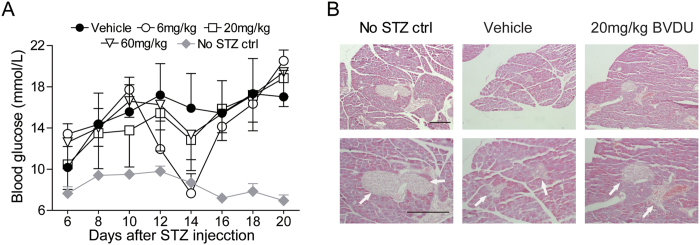
Therapeutic effects of BVDU on other autoimmune disease model. Type 1 diabetes was induced by STZ, fasted blood glucose was measured (**A**) and the tissue pathogenesis of pancreas and islet was analyzed by H&E staining (**B**). Scale bars: 200 μm.

As our data demonstrated that BVDU could inhibit IL-6, IL-12, IL-23 and IL-1β production, which participate in many other autoimmune diseases such as type 1 diabetes, we further analyzed the therapeutic effect of BVDU on STZ-induced type 1 diabetes. Blood glucose levels were analyzed after STZ injection, and the data showed that the blood glucose level in normal mice kept a relative stable low level of 8 mM (Fig. 7A, No STZ control group), however, the blood glucose was significantly elevated to a high level of 15 mM or higher level in mice injected with STZ (Fig. 7A, vehicle group), while this elevation of blood glucose level was inhibited upon BVDU treatment (Fig. 7A, 6-60 mg/kg groups), and the 20 mg/kg treatment group exhibited the best therapeutic effect, although the statistic was not significant, which might be attributed to the small mice numbers in each groups. Further histology analysis of pancreas showed that compared to vehicle group, both the pancreas structures and the islet volumes were almost restored to those of the normal mice upon BVDU treatment (Fig. 7B).

### BVDU reduces IL-6 production in primary human DC

**Figure 8:**
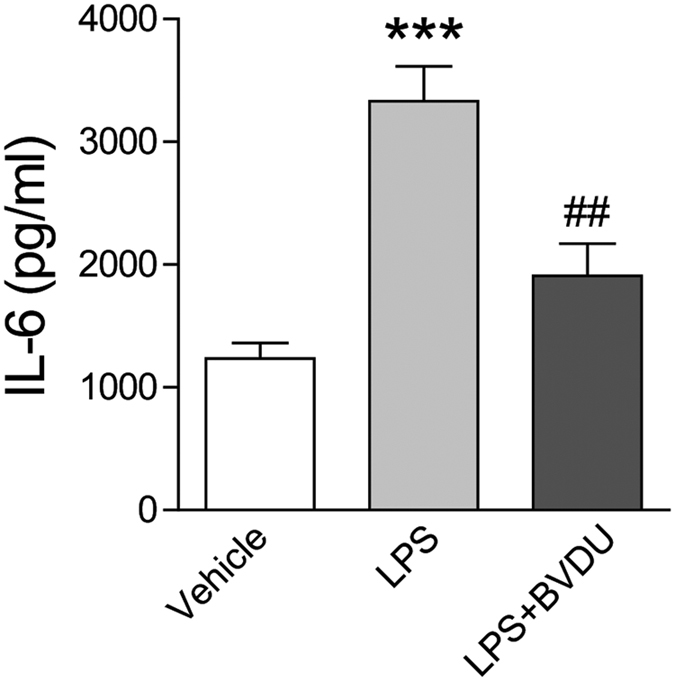
BVDU treatment reduces IL-6 production by primary cultured human DC. Human DC were isolated by CD1c antibody conjunct magnetic-beads, and incubated with RPMI 1640 with 10% FBS alone, or stimulated with LPS (100 pg/ml), or in combination with BVDU (10 μM) for 24 hours, the supernatants were collected for IL-6 production analysis by ELISA. Data are mean ± SEM (n = 3), ***p < 0.001 versus vehicle, ^##^*p* < 0.01 versus LPS treatment.

As our data demonstrated that BVDU treatment inhibited the IL-6 production by mouse DC cells, and we wondered that if this phenomena also existed in human DC, we isolated the primary DC from human periphery blood, and cultured for 72 h with LPS stimulation, the IL-6 production was detected in the culture supernatant, and we found that LPS stimulation boosted IL-6 production by human DC, while the BVDU treatment significantly inhibited the IL-6 production, this demonstrated that BVDU had similar function in regulating IL-6 production in human DC (Fig. 8).

## Discussion

As fundamental antigen presenting cells, DCs produce many cytokines which play major roles in activating T cells and inflammatory diseases. The attempt to regulate the cytokines secreted by DCs can provide a possibility to control many inflammatory diseases. To screen for drugs that target DCs, we could have harvested supernatant from cultured cells and used ELISA to measure cytokine contents and analyze the expression changes involved. But the process of performing an ELISA is complex, since we need to wash the plate for many times, this accompanied with a high cost which does not make it suitable for high throughput screening. HTRF is a newer technology to detect the sample in a pure liquid system; it combines the technology of FRET (Fluorescence Resonance Energy Transfer) and TRF (Time-Resolved Fluorescence). The operating process is simple, with only two steps, and without washing procedure. The detection system is stable and the cost is lower. With the above advantages, it’s widely used in drug screening recently. In our study, we found that there is a good match between the usage of HTRF and ELISA in IL-6 detection. By using this technology we found some drugs with known effects on IL-6 secretion in DCs.

During the screening process, we found that a few compounds decreased the IL-6 secretion not by preventing the ability of DCs to secret IL-6, but by reducing the DCs cell numbers because of their toxicity. So we decided to detect the cell numbers after drug treatment. Since it is laborious to count cells, we chose to take photos of GFP expressing cells. We use bone marrow cell derived from GFP transgenic mouse to generate DCs, and the supernatant was harvested for IL-6 detection while the cells were analyzed with GFP as a marker by measuring the number, size, and intensity of GFP spots with high-throughput fluorescent microscopy to decide the cell number after drug treatment. If the cell number was dramatically decreased then the IL-6 reduction was considered to be caused by drug toxicity.

Statins have cholesterol-lowering properties and are widely used in cardiovascular disease therapy. Increasing evidence indicates that Statins have immunomodulatory properties, via post translational protein modifications. In our study, we found that Statins have the ability to enhance IL-6 secretion, which is consistent with the report that Statins upregulate the secretion of proinflammatory cytokines by bone marrow derived dendritic cells induced by LPS but inhibit its maturation^24^. Vincristine, Colchicine, and Podophyllotoxin, are reported to have antitumor effects by inhibiting microtubule assembly in tumor cells, finally causing the death of tumor cells. However, recent evidence shows that these kind of anti-tumor drugs have pronounced immunomodulatory effects. A research provided by Hiroaki Tanaka shows that Vincristine promotes DCs maturation, enhances the expression of CD40, CD80, CD86 and MHCII, triggers the secretion of IL-1β, IL-6 and IL12p40, and enhances the ability of DCs to activate T cells. All of these were verified by our system. This goes to show that our system is sound and stable for selecting compounds that could affect DCs secreting IL-6, what’s more this system could be applied for large scale screening^15^.

In addition to the reported finding above, we also found that some other chemicals affected IL-6 secretion. Amiloride, a Na^+^/Ca^+^ exchange blocker, is a potassium-sparing diuretic that is widely used to treat hypertension and congestive heart failure. In our research, we found that Amiloride treatment inhibited DCs secretion of IL-6, reduced the mRNA expression of il12, il23, il1b, cd80 and cd86. There is a report that Amiloride reduced clinical deficit of EAE, providing neuroprotection^25^. We infer that the effect of Amiloride on DCs might be partially contributed to its curative effect in EAE.

BVDU is a potent inhibitor of herpes simplex virus type 1 (HSV-1) and varicella-zoster virus (VZV) infections, which was discovered 20 years ago, its mechanism is the specific phosphorylation of BVDU to its 5’-diphosphate (DP) by the virus-encoded thymidine kinase (TK); this compound interferes as a competitive substrate with the viral DNA polymerase. In our study, we found that BVDU decreased IL-6 secretion of DCs, down-regulated the mRNA expression of *il12, il23, il1b, tnfa, il10, cd80* and *cd86*, reduced the secretion of IL-12p70, IL-1β and alleviated DCs ability to activate Th17. LPS stimulation can lead to DCs maturation and the activation of MAPKs and NF-*κ*B is involved in DCs maturation and cytokine production^21^,^22^. LPS stimulation enhanced the phosphorylation of all three MAPKs including ERK, JNK, and p38 in DCs, which was absent in BVDU treatment. By detecting two components of NF-*κ*B, p50 and p65, we found a similar inhibition on NF-*κ*B of BVDU. We assumed that BVDU treatment suppressed MAPK and NF-*κ*B pathways in LPS stimulated BMDCs, which consequently led to suppressed IL-6 production. Finally, we performed *in vivo* animal experiments and the results showed a vast promising potential of BVDU for autoimmune diseases therapy. During EAE pathogenesis, our results showed that BVDU significantly reduced expression of IL-17A but not that of IL-17F and IL-22. Although IL-17A and IL-17F share the strongest sequence homology, the production regulation of IL-17A is distinct from that of IL-17F. For instance, PGE2 increases IL-17A concentrations but inhibits IL-17F production in B cell-T cell co-culture^26^. In addition, RORα is an important transcription factor of Th17 cell and its expression is IL-6/STAT3-dependent. It has been reported that RORα deficiency affects IL-17A expression rather than IL-17F or IL-22 expression, which is similar to what we found^27^. We supposed that BVDU suppression on IL-6 production might lead to decreased activation of STAT3 and consequently decreased expression of RORα, which preferentially regulated IL-17A production.

In summary, we reported a novel high-throughput screening assay on IL-6 production in DCs. By normalizing the assay results with GFP expression, we rendered the screening assay quite fast and convenient. BVDU was one of the results we got based on the screening assay. We found the therapeutic effects of BVDU on EAE and IL-6 level was also reduced after treatment of BVDU in mice model as well as in human samples, which was in accord with the cell screening model. With these *in vitro* and *in vivo* biological assays, we further validated the accurateness of our novel high-throughput screening assay and the potential applications of BVDU in autoimmune diseases.

## Materials and Methods

### Mice

C57BL/6 mice were purchased from Shanghai Laboratory Animal Center (Shanghai, China). All mice were housed in pathogen free condition with standard laboratory chow and water. All experiments were approved and conducted in accordance with the guidelines of the Animal Care Committee of Tongji University.

### *In vitro* generation of dendritic cells

Tibia and femur bones were removed from mice, ends of bones were clipped and marrow was flushed out with a syringe. Convert the marrow into single cell suspension and get rid of red blood cells with ACK lysing buffer. Cells were cultured in 10% FCS RPMI1640 (GIBCO) containing recombinant murine GM-CSF (Peprotech) at 20 ng/ml and recombinant murine IL-4 (Peprotech) at 1 ng/ml. On day 2 to 3, half of old medium was decanted, and half of freshly made medium containing the same concentration of GM-CSF and IL-4 was added. On day 6 or 7, blow the cells gently and collected non-adhered or loosely adhered cells.

### Flow cytometry

For surface staining of DCs, cells were harvested and resuspended at 5 × 10^5^/ml in PBS supplemented with 1% BSA. After washing, cells were incubated with fluorescence-labeled surface Abs against CD11c, CD80 or CD86 (eBioscience) for 30 min at 4 °C. Cells were then washed, resuspended in ice-cold FACS buffer and the the surface staining was analyzed. For intracellular staining of cytokines of splenocytes, cells were stimulated for 5 h at 37 °C with PMA (50 ng/ml), innomycin (750 ng/ml), and brefeldin A (3 μg/ml) (all from Sigma-Aldrich). After surface staining with fluorescence-labeled surface Abs against CD4, cells were resuspended in Fixation/Permeabilization solution (Cytofix/Cytoperm kit; BD Pharmingen) incubated with Abs against IL-17 (eBioscience) at 4 °C. A guava easyCyte 8HT flow cytometry system and guavaSoft (Millipore) were used for analysis.

### Reverse transcription and real-time PCR

Total mRNA was isolated using TRIzol reagent (Invitrogen) and1.5 μg RNA were used to synthesize cDNA using random hexamer primer and Moloney murine leukemia virus reverse transcriptase (Promega) according to the manufacturer’s protocol. Real-time PCR was performed using SYBR Green JumpStart^TM^ Taq ReadyMix^TM^ kit (Sigma) with LightCycler quantitative PCR apparatus (Stratagene) with the cycling program of 95 °C for 30 s, 60 °C for 30 s, and 72 °C for 30 s for 40 rounds. Results were normalized to β-actin expression in the same sample and then normalized to the control. The primer pairs used are shown in Supplementary Data 1.

### Enzyme-linked immunosorbent assay (ELISA) and cytokine measurement

DCs were cultured into 96-well plates at a density of 10,000 cells per well with different condition for 24–36 hours, or plant at different cell numbers for the sake of optimize of cell numbers for screening system. The culture medium was collected, clarified by centrifugation for 10 min at 14,000 × g, and the concentration of IL-6, IL-12p70, IL-1β was measured using Mouse ELISA kits (DAKEWE) according to the manufacturer’s protocol.

### Establish and optimize of the screening system

BMDCs were seeded into 96-well plates (Corning) at 10,000 cells per well in 10% FCS RPMI 1640 containing GM-CSF at 20 ng/ml and IL-4 at 1 ng/ml followed by incubation at 37 °C for 1 hour to allow for plate adherence. Compounds were transferred into duplicate plate. For each treatment plate, 8 of 96 wells were added with DMSO (1%) as negative control, 4 of 96 were added with 5′-(N-Ethylcarboxamid) adenosine (NECA, 10 μM final concentration) as positive control, and 4 of 96 were added with Dexamethasone (DEX, 100 nM final concentration) as another positive control. 1 hour after compound treatment, BMDCs were stimulated with LPS (10 ng/ml final concentration) dispersed in culture medium. After 24 hours, 10 μl of resulting supernatant was transferred from the culture plate to 384-well plates (PerkinElmer). IL-6 abundance in the supernatant was determined using Mouse IL-6 assay kit (Cisbio) according to the manufacturer’s protocol and signal intensity measured using an envision multimode plate reader (PerkinElmer).

### DC-T cell co-culture

Spleens were harvested from C57BL/6 mice and CD4^+^ T cells were enriched by negative CD4^+^ T cell isolation kit (Invitrogen). Purified CD4^+^ T cell (100,000 cells/well) added with anti-CD3 (2 μg/ml) and anti-CD28 (2 μg/ml) antibodies were co-cultured with DCs (10,000 cells/well) pretreated with BVDU or not. For Th17 differentiation, anti-IFN-γ (10 μg/ml), anti-IL-4 (10 μg/ml), recombinant human TGF-β1 (2 ng/ml), rmTNF-α (10 ng/ml), rmIL-23 (10 ng/ml), and IL-1β (10 ng/ml) were added. Three days later, the proliferation of CD4^+^ T cells was analyzed by flow cytometry.

### Western blot

BMDCs were treated with LPS in the presence of BVDU or not for the indicated duration at 37 °C, then cells were lysed, sonicated and boiled at 95 °C for 5 min in sample buffer. BMDCs lysates were separated on SDS-PAGE on 10% polyacrylamide gels and transferred to polyvinylidene fluoride (PVDF) membranes. Then the PVDF membranes were incubated with blocking buffer containing 5% non-fat milk for 1 h and later incubated overnight at 4 °C in buffer containing antibody against ERK/P-ERK (CST), JNK/p-JNK (CST), p38/p-p38 (CST), NF-*κ*Bp50, p65 (SantaCruz) or GAPDH (CST). After washing for three times, the membranes were incubated with proper HRP-conjugated secondary antibodies for 1 hour. Later after washing thrice, immunostaining was visualized using Western Lightning Ultra (PerkinElmer).

### EAE induction and BVDU treatment

Mice of 8–10 weeks of age were s.c. immunized with 200 μg myelin oligodendrocyte glycoprotein (MOG)_35–55_ (MEVGWYRSPFSRVVHLYRNGK; obtained from GL Biochem) in CFA (Sigma-Aldrich) containing 5 mg/ml heat-killed Mycobacterium tuberculosis H37RA (Difco Laboratories). 200 ng of pertussis toxin (Calbiochem) was injected i.p. to each mouse on days 0 and 2. Mice were assigned scores on the following scale: 0, no clinical signs; 1, tail weakness; 2, hind limb weakness; 3, paraplegia; 4, paraplegia with forelimb weakness or paralysis; 5, moribund or dead. For drug treatment, mice received 20 mg/kg of BVDU by administered orally once daily from day 3 to day 24. Saline was given as a vehicle control (200 μl for each mouse).

### STZ-induced type 1 diabetes

STZ was dissolved in the Na-Citrate buffer (pH 4.5), and used within 20 min of preparation. Mice were fasted prior to injection. STZ was injected i.p. into mice (50 mg/kg) for 5 days consecutively. Fasted blood glucose was measured by Accu-Chek Performa (Roche Diagnostics). Blood glucose levels of >13.9 mM were considered to be diabetic.

### Histology analysis

Mice were anesthetized and perfused with PBS (pH 7.4) followed by 4% (w/v) paraformaldehyde. Spinal cords or pancreas samples were collected and fixed in 4% (w/v) paraformaldehyde overnight. Paraffin-embedded sections of spinal cord and pancreas were stained with H&E or luxol fast blue.

### Human DCs isolation and IL-6 production analysis

PBMCs were isolated from the blood of healthy donors by density centrifugation using Lymphoprep^TM^ (STEMCELL). DCs were collected by CD1c^+^ (BDCA-1) Cell Isolation Kit (MiltenyiBiotec). Freshly isolated DCs (1 × 10^5^) were incubated in 200 μl RPMI 1640 with 10% FBS for 24 hours alone, or in combination with LPS (100 ng/ml) in the presence of BVDU (10 μM) or not, and IL-6 production in the supernatant was measured by Human IL-6 ELISA Ready-SET-Go (eBioscience). Human blood samples were obtained from healthy volunteers from Tongji University. Informed consent was provided, and the sampling was completed in accordance with the guidelines of local institutional review boards. All of the experimental protocols were approved by the ethical board of Medical School, Tongji University.

### Statistical analysis

Data were analyzed with Graphpad Prism software. Data are presented as means ± SEM. The statistical significance of the EAE clinical scores between treatments was analyzed with a two-way ANOVA test. Other analyses were assessed by a Student *t* test. A *p* value < 0.05 was considered statistically significant.

## Additional Information

**How to cite this article:** Chen, S. *et al*. Discovery of BVDU as a promising Drug for autoimmune diseases Therapy by Dendritic-cell-based functional screening. *Sci. Rep.* **7**, 43820; doi: 10.1038/srep43820 (2017).

**Publisher's note:** Springer Nature remains neutral with regard to jurisdictional claims in published maps and institutional affiliations.

## Supplementary Material

Supplementary Information

## Figures and Tables

**Table 1:** Drugs enhanced IL-6 secretion in mDCs.

No.	Drug name	Drug target	Cell number (folds of DMSO)	Reported Specific Cell lines or animal model associate with IL-6 production
7-B9	Formoterol	beta2-Adrenoceptor agonist	0.71	Upregulates IL-6 secretion in human airway smooth muscle cells^28^.
10-D5	Mevastatin	Antibiotic; inhibits post-translational prenylation of proteins such as Ras and geranylgeranylation of Rho	1.02	The same family member Lovastatin upregulates bone marrow-derived dendritic cell proinflammatory cytokine production^24^.
7-D9	Fluspirilene	Dopamine receptor antagonist; antipsychotic	0.66	
1-B7	YM 976	Phosphodiesterase type IV (PDE4) inhibitor. Exhibits anti-inflammatory activity without emesis.		
5-E3	Cetirizine dihydrochloride	Orally active, non-sedating, and selective H1 histamine receptor antagonist; antihistamine.	0.79	
2-A5	Adenosine 3′,5′-cyclic monophosphate	PKA activator; second messenger.	0.98	Synergistic induction of IL-6 expression by endothelin-1 and cAMP in adipocytes^29^. cAMP/PKA enhances IL-6 synthesis through STAT3 in glial cells^30^.
4-F5	2-Chloroadenosine	Adenosine receptor agonist with selectivity for A1 over A2	1.147	Increases IL-6 production by human gingival fibroblasts without any other stimuli^31^. 2-chloroadenosine increases IL-6 secretion and gene transcription in primary astrocytes^32^.
11-B6	Metrifudil	Adenosine receptor agonist which displays some selectivity for the A2 receptor type	1.01	
12-E9	Podophyllotoxin	Antineoplastic glucoside; inhibitor of microtubule assembly	1.02	Upregulates bone marrow-derived DCs IL-6 production by trigging NFκB activation^14^.
4-G11	Colchicine	Prevents tubulin polymerization	1.14	Upregulates bone marrow-derived DCs IL-6 production via NFκB pathway^14^.
16-C8	Vinblastine sulfate salt	Inhibitor of microtubule assembly	1.30	Induces the production of IL-6 and IL-12; increases CD40, CD80, CD86 and MHCII in DCs^33^.
16-A9	Vincristine sulfate	Inhibitor of microtubule assembly	1.23	

**Table 2:** Drugs decreases IL-6 secretion in mDCs.

No.	Drug name	Drug target	Cell number (folds of DMSO)	Reported Specific Cell lines or animal model associate with IL-6 production
14-G9	Sanguinarine chloride	Inhibitor of Mg^2+^ and Na^+^/K^+^ -ATPase; isolated from the leaves and stems of Macleayacordata and microcarpa	0.92	Sanguinarine decreases LPS-induced il6 gene expression in THP-1 cell line^34^.
2-C5	5-(N,N-hexamethylene) amiloride	Na^+^/H^+^ antiport inhibitor	0.98	
1-E6	5-(N-Ethyl-N-isopropyl) amiloride	Selective blocker of Na^+^/H^+^ antiport		
1-E9	5-(N-Methyl-N-isobutyl) amiloride	Potent blocker of the Na^+^/H^+^ antiport		
3-E10	ML-9	Myosin light chain kinase (MLCK) inhibitor	0.80	The same family member ML-7 decreases IL-6 protein level in colonic mucosa during inflammatory bowel disease pathogenesis^35^.
3-B6	(E)-5-(2- Bromovinyl)-2′- deoxyuridine	Potent inhibitor of herpes simplex type 1	0.84	
4-E11	Z-L-Phechloromethyl ketone	Bovine chymotrypsin A-gamma inhibitor	1.15	
8-F10	CPNQ	Prevents huntingtin-mediated proteosome dysfunction and reduces alpha-synuclein-mediated toxicity; promotes inclusion formation in cellular models of Huntington’s and Parkinson’s disease.	0.77	
7-E8	Flunarizine dihydrochloride	Ca^2+^/Na^+^ channel blocker; vasodilator	1.01	
