# Supplementary material for: Discovery of BVDU as a promising Drug for autoimmune diseases Therapy by Dendritic-cell-based functional screening
Source: Sci Rep. 2017 Mar 8;7:43820. doi: 10.1038/srep43820 (PMC5341058; doi:10.1038/srep43820)

Discovery of BVDU as a promising Drug for autoimmune diseases Therapy by Dendritic-cell-based functional screening

Shuai Chen[1], Jinfeng Zhou[1], Yingying Cai[1], Xinyuan Zheng[1], Sirong Xie[1], Yuhan Liao[1], Yu Zhu[1], Chaoyan Qin[1], Weiming Lai[1], Cuixia Yang[1], Xin Xie[2,3], Changsheng Du[1,3*]

[1]Department of Central Laboratory, Shanghai Tenth People's Hospital of Tongji University, School of Life Sciences and Technology, Tongji University, Shanghai 200092, China.

[2]State Key Laboratory of Drug Research, Shanghai Institute of Materia Medica, Chinese Academy of Sciences, Shanghai 201203, China.

[3]Shanghai Key Laboratory of Signaling and Disease Research, School of Life Sciences and Technology, Tongji University, Shanghai 200092, China.

[*]Address correspondence to: Dr. Changsheng Du, 1239 Si Ping Road, Shanghai 200092, China; Fax: 0086-21-65982579; E-mail: ducs2015@163.com

Supplementary information

Supplementary table.The sequences of the primer pairs for real-time PCR.

Gene	Sense primer (5' to 3')	Anti-sense primer (5' to 3')
cd11c	CAAGAAGCACCGAACATGGTT	GTCTGAGCTAGAGTCACTGGT
cd80	ACCCCCAACATAACTGAGTCT	TTCCAACCAAGAGAAGCGAGG
cd86	CTGGACTCTACGACTTCACAATG	AGTTGGCGATCACTGACAGTT
il6	ACCACGGCCTTCCCTACTTC	GAATTGCCATTGCACAACTCTT
il10	GCTCTTACTGACTGGCATGAG	CGCAGCTCTAGGAGCATGTG
il23	CTCTACTCCCTGATAGCCCCAT	TGCTCCGTGGGCAAAGAC
tnfα	CTCAAAATTCGAGTGACAAGCCT	GGTTGTCTTTGAGATCCATGCC
il12	CCCTGTGCCTTGGTAGCATC	GCGCAGAGTCTCGCCATT
il1b	AAGCCTCGTGCTGTCGGA	CAGGGTGGGTGTGCCGT
il17a	TTAACTCCCTTGGCGCAAAA	CTTTCCCTCCGCATTGACAC
il17f	TGCTACTGTTGATGTTGGGAC	AATGCCCTGGTTTTGGTTGAA
il22	GTGAGAAGCTAACGTCCATC	GTCTACCTCTGGTCTCATGG
tgfb	CTCCCGTGGCTTCTAGTGCT	AGCCTTAGTTTGGACAGGATCTG
b-actin	GGCTGTATTCCCCTCCATCG	CCAGTTGGTAACAATGCCATGTT

Supplementary Figure.BVDU treatment reduces neuronal damage in CNS of EAE mice. Immunofluorescence staining of the GAFP (gliosis), NFH (neurofilament) and DAPI in the paraffin sections of spinal cords isolated from EAE-induced mice treated with BVDU or vehicle on day 24 PI. Scale bars, 200μm.

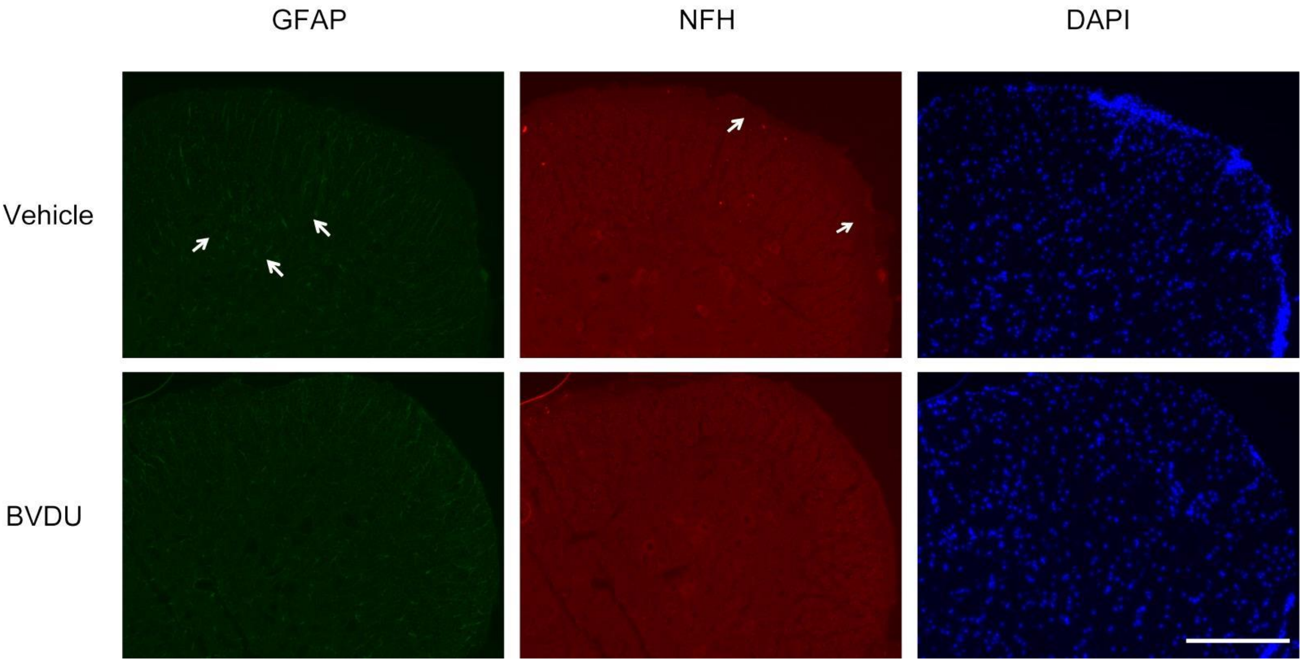

Supplement: Supplementary Information [file srep43820-s1.pdf]
